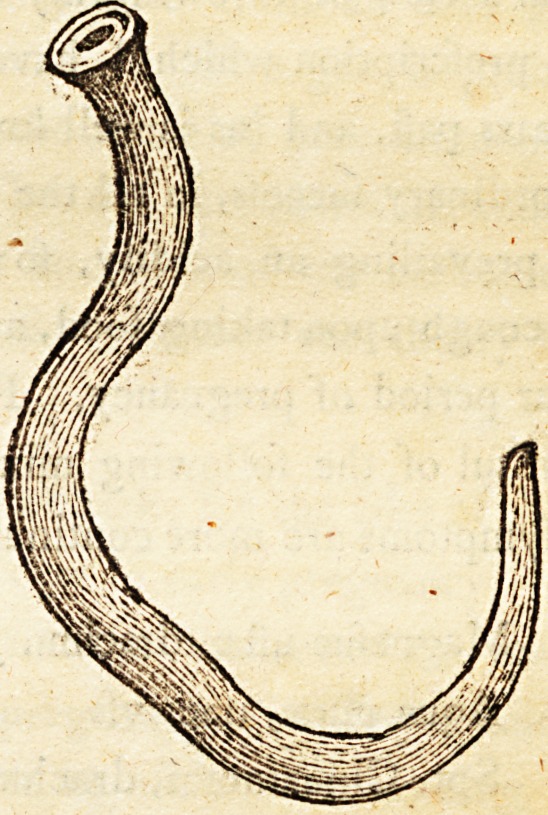# Dr. Marshal and Dr. Wiesenthal, on Worms Found in Poultry

**Published:** 1799-10

**Authors:** Andrew Marshal

**Affiliations:** Bartlet's-Buildings


### *To the Editors of the Medical and Physical Journal.*

Gentlemen,

I send you the following extract of a letter from an ingenious physician, Dr. A. Wiesenthal, Professor of Anatomy, at Baltimore, in North America; if you think as I do, that the communication is curious and interesting, you will allow it a place in your instructive monthly publication.

I am respectfully, Gentlemen,

Your most obedient and humble servant,

ANDREW MARSHAL.

Bartlet's-Buildings,

*Sept.* 10, 1799.

---

" Baltimore, *in* Maryland, *May* 21, 1797.

" There is a disease prevalent among the gallinaceous poultry in this country, called the *gaps,* which destroys eight tenths of our fowls in many parts, and takes place in the greatest degree among the young turkeys and chickens bred upon old established farms. I know not whether the same kinds of fowls in England are liable to it, and therefore shall take the liberty to give you a brief account of it.

" Chicks and poults, in a few days after they are hatched, are found frequently to open their mouths wide, and gasp for breath, at the same time frequently sneezing, and attempting to swallow. At first the affection is slight

ſlight, but gradually becomes more and more oppreſſive, until it ultimately deſtroys. Very few recover; they languiſh, grow diſpirited, droop, and die. It is generally known, that theſe ſymptoms are occaſioned by worms in the trachea. I have ſeen the whole of it completely filled with theſe worms, and have been aſtoniſhed at the animal's being capable of reſpiration under ſuch circumſtances. The annexed cut is a repreſentation of theſe animalculæ of the natural figure, and magnified.

"The ſmall figure repreſents the worms of their natural ſize, found in the trachea of chickens and young turkeys: the large figure, the ſame magnified. They are of a reddiſh colour, and at firſt view, reſemble the human *lumbricus;* but when examined, are materially different. When expoſed to the microſcope, they are found to have an orifice or mouth at one end, formed for ſuction; the other end, as far as I know, imperforated. Through the integuments is ſeen the inteſtinal tube, much convoluted, like that of the *lumbricus.*

"No effectual remedy is known againſt theſe moſt deſtructive animals. I have indeed ſeen them drawn out of the trachea, by means of a feather ſtripped from near its end, which is paſſed into the larynx, and twiſted round till it engages one or two of the worms, which are extracted with it.

"ANDREW WIESENTHAL."

## Figures and Tables

**Figure f1:**
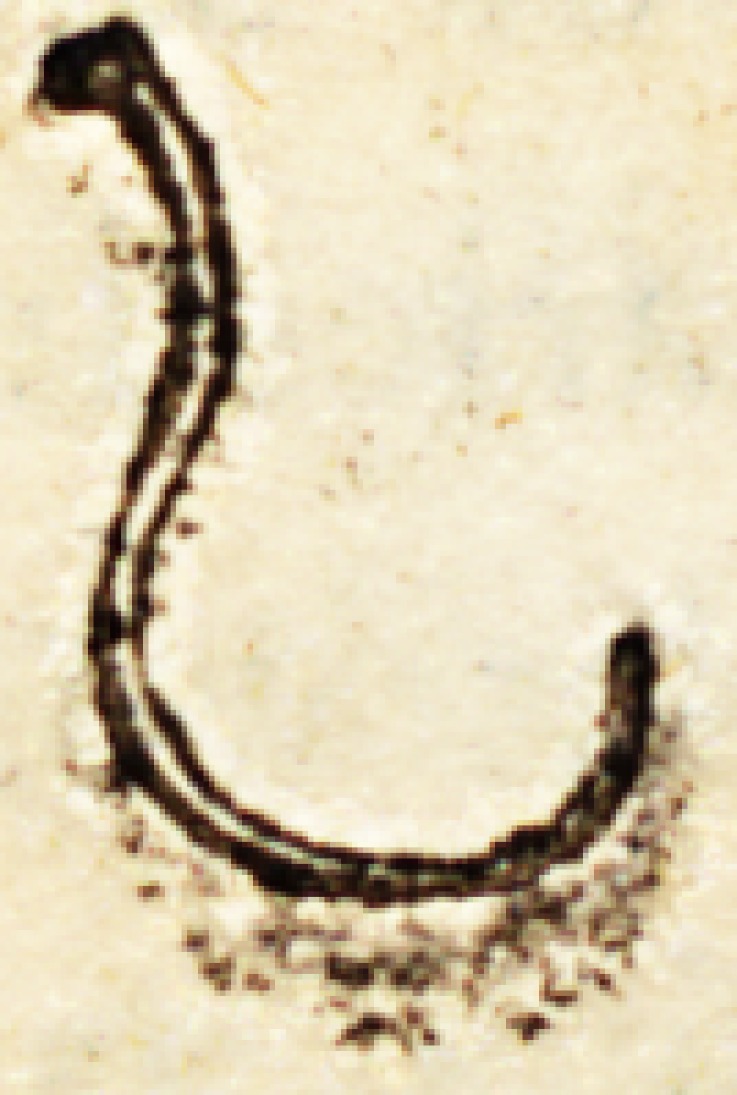


**Figure f2:**